# The Work Engagement Among Nurses in an Urban-Based Tertiary Hospital

**DOI:** 10.3390/nursrep15070241

**Published:** 2025-07-01

**Authors:** Ampan Vimonvattana, Nontawat Benjakul

**Affiliations:** 1 Division of Strategy and Quality Improvement, Faculty of Medicine Vajira Hospital, Navamindradhiraj University, Bangkok 10300, Thailand; ampan@nmu.ac.th; 2 Department of Anatomical Pathology, Faculty of Medicine Vajira Hospital, Navamindradhiraj University, Bangkok 10300, Thailand; 3 Health Administration Target Research Interest Group, Faculty of Medicine Vajira Hospital, Navamindradhiraj University, Bangkok 10300, Thailand

**Keywords:** work engagement, nurses, urban hospital, utrecht work engagement scale, nursing administration, nursing management, healthcare workforce

## Abstract

**Background**: Work engagement is essential to the well-being of nurses and the quality of health care, particularly in high-demand urban hospital environments in Bangkok. To determine the levels of work engagement—vigor, dedication, and absorption—among nurses in a Thai urban tertiary hospital, and to identify associated demographic and occupational predictors. **Materials and Methods**: A cross-sectional study was conducted among 650 nurses at a tertiary university hospital in Bangkok, Thailand, from February to March 2025. Participants were selected through simple random sampling. They completed an online survey including demographic data and the Utrecht Work Engagement Scale (UWES), which assesses three dimensions of engagement: vigor, dedication, and absorption. To identify the predictors of high engagement levels, chi-square tests and multivariate binary logistic regression were used. **Results**: Most nurses reported low engagement across all dimensions: 73.1% for vigor, 69.1% for dedication, and 70.0% for absorption. In the adjusted models, monthly income was a significant predictor of higher vigor and dedication, whereas no significant predictors emerged for absorption. Other variables, including age, experience, and professional rank, were significant in the bivariate analyses but not in the multivariate models. **Conclusions**: Nurse engagement remains suboptimal in the urban tertiary hospital setting, with financial compensation emerging as a key determinant. Strategic interventions to improve income equity and career development may help enhance engagement and retention in the nursing workforce.

## 1. Introduction

Globally, the nursing workforce faces critical challenges, with the WHO estimating a shortfall of 5.9 million nurses. Retention strategies are therefore essential. Reports from the United Kingdom, the United States of America, and other regions highlight burnout, lack of career progression, and poor workplace support as leading causes of attrition [1]. The nursing shortage crisis has had a significant national impact. The turnover rate of professional nurses has risen annually [2], accompanied by an increase in their workload [3]. This has resulted in accumulated stress and fatigue, which have an adverse effect on the well-being of professional nurses [4]. If not managed properly, this situation may lead to burnout and subsequent resignation. Conversely, professional nurses who remain employed may retain a certain level of affiliation with the organization [5]. To effectively address this issue, it is essential to evaluate the work commitment of professional nurses using suitable measurement instruments [6]. Assessing professional nurses’ work engagement using suitable instruments can have a great influence on management planning.

Work engagement is a concept that refers to individuals’ motivation to commit themselves to their work with complete capacity, both physically and mentally [7,8]. Individuals who demonstrate vigor (i.e., high levels of energy and mental resilience while working, the willingness to invest effort in one’s work, and persistence even in the face of difficulties), dedication (characterized by a sense of significance, inspiration, pride, enthusiasm, and challenge), and absorption (which refers to applying full concentration and being engrossed in one’s work) toward their tasks are more likely to be highly engaged [9,10,11]. These individuals can still surmount obstacles or difficulties even in challenging situations or under pressure [12]. Moreover, research on the determinants of work engagement has revealed that a high quality of working life is positively associated with enhanced work engagement [13,14].

Schaufeli and colleagues developed the Utrecht Work Engagement Scale (UWES) [15], which assesses work engagement. The UWES is widely used in Thailand and has been validated as a reliable and comprehensive tool for evaluating the professional context of nurses in both the private [16] and public sectors under the Ministry of Public Health [17].

The assessment of professional nurses’ work engagement will aid in the formulation of operational policies for nurses, aligning with the objectives and functions of tertiary hospitals, which are health care institutions that deliver specialized medical services with optimal efficiency. While the determinants of engagement have been widely studied internationally, data from Southeast Asia—and particularly Thailand—remain sparse. Numerous systematic reviews and meta-analyses have examined nurse work engagement globally, revealing strong associations between engagement and patient outcomes, leadership styles, and personal resources such as resilience and psychological capital. Some studies [18,19,20] emphasize that both organizational factors (e.g., leadership support, work environment) and individual psychological resources are critical determinants. However, there remains a paucity of evidence about how these factors manifest in urban, tertiary-care hospitals in Thailand. Understanding this gap is essential for tailoring engagement strategies in this specific healthcare context.

## 2. Materials and Methods

We conducted a cross-sectional study that collected data from a tertiary urban-based university hospital in Bangkok, Thailand. Data were gathered from February 2025 to March 2025, and the survey was sent to nurses through the individual emails of the Faculty of Medicine Vajira Hospital, Navamindradhiraj University. We ensured the privacy and anonymity of the participants during the data collection process, adhered to established practices in this research, and identified no ethical issues.

We selected participants using a simple random sampling technique from the nursing department of Vajira Hospital. Participants completed an online questionnaire through a self-reported survey created on Google Forms. Before data collection, we obtained written online consent from the participants. By disabling all Google Form settings that collect email addresses or internet protocol address information. We ensured participant anonymity. All responses were anonymous and unlinkable to individuals, even when demographic data were collected.

The questionnaire consisted of the following three sections. The first section focused on establishing the eligibility criteria for the research participants, comprising four closed-ended questions for selection. The second section collected general information regarding the participant, including sociodemographic information such as gender, age group, monthly income, monthly income adequacy, years of work experience, working division, and position levels. This section used closed-ended questions for selection, culminating in a total of seven questions. The third section of the questionnaire examined the factors affecting the UWES [15], consisting of six questions for vigor assessment, five questions for dedication assessment, and six questions for absorption assessment.

The UWES assesses three dimensions of engagement: vigor—high energy and resilience at work, dedication—strong involvement and a sense of significance, and absorption—being fully concentrated and engrossed in work. The Thai version of UWES used in this study has undergone psychometric validation and cultural adaptation in a previous study [15,16]. This section was evaluated using a 7-point Likert scale (range, 1 to 7).

The categorization of Likert scale scores was guided by Thai validation studies [15,16] that used the 75% threshold for a high level and explained its application in line with regional research practices. Finally, we categorized the scores as low (<32) and high (≥32) for vigor, low (<27) and high (≥27) for dedication, and low (<31) and high (≥31) for absorption. In this study, we coded the responses for the dependent variable as 0 = low and 1 = high.

The independent variables were the demographic characteristics of the participants. The demographic and clinical characteristics included in this study were guided by previous disability studies including gender (female, male), age group (years) (20 to 30 [Generation Z], 31 to 45 [Millennials], 46 to 60 [Generation X]), monthly income (Baht) (≤20,000, 20,000 to 40,000, 40,001 to 60,000, >60,000), adequacy of monthly income (not enough, enough), years of work experience (1 to 10, 11 to 20, 21 to 30, 31 to 40), working division (executives and system administrators, outpatient department, inpatient department, intensive care unit, special clinic unit, operating department, anesthesia department, emergency department), position level (practitioner level, professional level, senior professional level).

We used descriptive statistics to summarize the characteristics of the participants. We tested the bivariate associations between the independent variables and the UWES levels using the chi-square test. We entered variables that showed statistically significant associations in the bivariate analysis into a multivariate binary logistic regression model to identify the predictors of vigor, dedication, and absorption. The final model for vigor and dedication included age group, monthly income, monthly income adequacy, years of work experience, and position levels. The final model for absorption included the age group, years of work experience, and position level. We selected these variables based on their theoretical relevance and statistical significance in the preliminary analysis.

## 3. Results

Table 1 shows the demographic characteristics of the 650 participating nurses. Most participants were female (95.2%). In terms of age distribution, the largest proportion belonged to Generation X (41.8%), followed by Millennials (32.6%). Nearly half of the participants reported a monthly income between 20,000 and 40,000 Baht (48.3%), whereas only 8.8% earned more than 60,000 Baht. With regard to perceived income adequacy, 58.0% indicated their income was sufficient, whereas 42.0% reported it was inadequate. Most had 1 to 10 years of work experience (53.2%), and the most common workplace setting was the inpatient department (37.8%), followed by the intensive care unit (20.5%). In terms of position rank, most participants were at the practitioner level (60.9%), followed by professionals (29.9%).

Table 2 summarizes the descriptive statistics for each dimension of the UWES, including the minimum, maximum, mean, standard deviation, and the proportion of respondents classified into high- and low-engagement categories. The mean scores (± standard deviation) were as follows: vigor = 27.90 ± 6.24, dedication = 24.24 ± 5.43, and absorption = 27.29 ± 6.67. Most participants fell into the low-engagement category across all three dimensions, with 26.9% scoring a high level on vigor, 30.9% on dedication, and 30.0% on absorption.

We explored the association between demographic characteristics and levels of vigor, as shown in Table 3. We found statistically significant associations with age (*p* = 0.006), monthly income (*p* = 0.033), adequacy of monthly income (*p* = 0.039), years of work experience (*p* = 0.004), and position level (*p* = 0.001). Nurses with higher income adequacy, longer work experience, and a higher professional rank were more likely to report high levels of vigor.

Table 4 shows the dedication scores in relation to the demographic variables. We noted significant differences for age groups (*p* < 0.001), monthly income (*p* < 0.001), income adequacy (*p* = 0.050), years of experience (*p* < 0.001), and position level (*p* < 0.001). Younger nurses (particularly Generation Z), those with adequate income, and those in senior professional roles exhibited significantly higher levels of dedication.

We investigated the correlation between demographic factors and absorption, and the results are shown in Table 5. Age (*p* < 0.001), years of work experience (*p* < 0.001), and position level (*p* < 0.001) demonstrated statistically significant associations. Notably, a higher proportion of nurses in the senior professional group exhibited high absorption levels, whereas gender, monthly income, and monthly income adequacy were not significantly related to absorption scores.

Table 6 presents the multivariate binary logistic regression analysis for vigor. After adjusting for potential confounders, monthly income remained a significant predictor. Nurses earning 20,000 to 40,000 Baht (adjusted odds ratio [AOR] = 0.478, 95% confidence interval [CI]: 0.28–0.83, *p* = 0.009), 40,001 to 60,000 Baht (AOR = 0.414, 95% CI: 0.20–0.87, *p* = 0.019), and greater than 60,000 Baht (AOR = 0.308, 95% CI: 0.11–0.87, *p* = 0.026) were significantly less likely to report high levels of vigor compared with those earning ≤ 20,000 Baht.

Table 7 illustrates the multivariate logistic regression analysis for dedication. Monthly income was again a significant predictor; nurses earning 20,000 to 40,000 Baht were less likely to report high dedication scores (AOR = 0.533, 95% CI: 0.31–0.91, *p* = 0.021) than those in the lowest income bracket. Other demographic variables, including age, work experience, and position level, did not retain statistical significance in the adjusted model.

Table 8 displays the multivariate logistic regression findings for absorption. None of the included demographic variables were significant predictors in the adjusted model. Although the AORs for age, years of experience, and position level suggested possible associations, all *p-values* exceeded the threshold for significance.

## 4. Discussion

Numerous systematic reviews and meta-analyses have investigated nurse work engagement, consistently demonstrating its critical role in patient safety, quality of care, and nurse retention. For instance, the pandemic reported a marked decline in nurse engagement globally due to heightened work demands and emotional strain [18]. The significant positive associations between work engagement and patient care quality suggest that engaged nurses contribute to better patient outcomes [19]. Additionally, leadership styles have been shown to influence engagement substantially [19,20]. Our findings of low engagement levels align with these global trends and underscore the urgent need for context-specific strategies to improve engagement in Thailand’s urban tertiary hospitals.

This study investigated the levels of work engagement—vigor, dedication, and absorption—among nurses employed in an urban-based tertiary hospital and identified demographic and occupational factors associated with engagement. The finding that most participants reported low levels of engagement is consistent with several global and regional studies. For instance, similarly low engagement among nurses worldwide during the pandemic is attributed to increased job demands and emotional stress [18,21]. Moreover, some studies revealed moderate-to-low engagement levels, often linked to leadership styles, work environment, and cultural expectations [20,21,22,23]. Compared to these studies, our results reflect similar challenges but highlight unique factors, such as the inverse relationship between higher income and engagement, which warrants further exploration in the Thai context.

Our findings revealed that most participating nurses reported low levels of engagement across all dimensions, which was consistent with a prior study [24]. Specifically, only 26.9%, 30.9%, and 30.0% of participants demonstrated high levels of vigor, dedication, and absorption, respectively. These results raise concerns regarding the current state of motivational and psychological well-being among professional nurses, even in a well-resourced, university-affiliated hospital.

The multivariate analyses identified monthly income as a significant predictor of both vigor and dedication. In contrast to conventional assumptions, our results revealed that nurses in higher income brackets were significantly less likely to report high levels of vigor and dedication. This counterintuitive finding may reflect a complex interplay of expectations, work-life balance, and intrinsic versus extrinsic motivation. Lower-income nurses may derive engagement from job stability, professional identity, or altruistic values, whereas higher-income earners may face increased administrative burdens or role strain, which could dampen their engagement [25]. This finding supports the existing evidence that adequate compensation functions as a key resource, enabling health care professionals to invest more energy, commitment, and focus in their roles. Although other variables such as age group, years of experience, and position level were significantly associated with engagement in the bivariate analyses, these did not remain statistically significant in adjusted models, suggesting that income-related variables may play a mediating role in the relationship between demographic factors and engagement.

The inverse correlation we observed between income and engagement contrasts with findings from some international studies, which often report higher engagement among better-compensated nurses [22,23]. However, there are complex correlations between income, leadership styles, and engagement levels, suggesting that income alone does not predict engagement in all contexts. These inconsistencies highlight the need for culturally sensitive interpretations and interventions tailored to local realities [19,20,21,22,23]. In contrast with our expectations, younger nurses (Generation Z) did not demonstrate significantly higher engagement levels than their older counterparts did in the multivariate analyses. This indicates that generational differences alone do not sufficiently explain the variance in engagement [26,27,28]. Instead, work-related factors such as role expectations, professional support, and institutional recognition may exert a stronger influence [29].

The Job Demands–Resources model, which posits that work engagement is the result of a balance between professional demands and available personal or organizational resources, is consistent with these findings [30,31]. In the context of tertiary hospitals, where the workload is typically high, the presence of sufficient resources—including fair compensation, career advancement, and supportive environments—is crucial for sustaining nurse engagement [32,33]. This finding aligns with the Job Demands–Resources (JD-R) model, which underscores the collaborative impact of job demands (e.g., workload, emotional exhaustion) and job resources (e.g., compensation, autonomy, support) on engagement. Although income is generally regarded as a resource, its influence may be influenced by the demands that are associated with it. In this study, increased income may be associated with increased responsibilities, thereby transitioning it from a motivational resource to a potential job demand.

Despite its contributions, this study has several limitations. First, the cross-sectional design precludes causal inference; it remains unclear whether higher income leads to greater engagement or whether more engaged individuals pursue higher paying roles. Second, the use of self-reported online surveys may introduce response biases, such as social desirability bias or selective participation. Third, we conducted the study in a single tertiary-care hospital, which might limit the generalizability of the results to other health care settings, particularly rural or secondary-level institutions. Fourth, although we considered key demographic and occupational variables, other important factors—such as leadership support, psychological capital, work–life balance, and team dynamics—were not assessed and might have influenced engagement levels. Lastly, we cannot rule out the possibility of nonresponse bias, as highly disengaged nurses might have opted not to participate. Furthermore, the study did not incorporate organizational variables such as leadership style, teamwork climate, autonomy, or psychological safety. These factors are well-documented in the literature as crucial determinants of engagement and should be prioritized in future models.

Based on the findings and limitations, we can propose several recommendations. Hospital administrators should consider implementing financial incentive structures and equitable compensation models to address income-related disparities in engagement [31,34]. In addition, career development programs and clear pathways for professional advancement may help foster a greater sense of dedication and fulfillment, particularly among junior and mid-career nurses [33,35]. Institutions should also consider integrating regular assessments of engagement into their workforce evaluation strategies using validated tools such as the UWES. This would enable the early identification of disengaged groups and the development of targeted interventions [36,37]. Future research should adopt longitudinal designs to explore causal relationships and include broader organizational, psychosocial, and contextual variables to develop a more comprehensive understanding of the factors that influence nurse engagement.

Finally, this study highlights the need for multilevel strategies—ranging from financial to professional support—to enhance and sustain work engagement among nurses in high-demand urban hospital settings. Addressing these issues is essential to ensure workforce stability, patient care quality, and institutional resilience in the face of health care system challenges.

## 5. Conclusions

In this study, we revealed that most nurses in a tertiary urban hospital reported low levels of work engagement across the dimensions of vigor, dedication, and absorption. Among the factors examined, monthly income emerged as a significant predictor of engagement, particularly in relation to vigor and dedication. The findings underscore the importance of financial and professional support in fostering nurse engagement in high-demand health care environments. Despite working in a university-affiliated institution, many nurses exhibited signs of diminished motivation, highlighting systemic challenges that extend beyond individual capacity.

To enhance nurse engagement, hospital administrators and policymakers should prioritize interventions that improve income equity, career development, and supportive work environments. The regular monitoring of engagement levels using validated instruments can aid in the early identification of at-risk groups and inform tailored strategies. To provide a more comprehensive understanding of the determinants of work engagement in the nursing workforce, future research should use longitudinal designs and incorporate organizational and psychosocial factors.

Ultimately, strengthening work engagement is essential not only for the well-being and retention of nurses but also for the delivery of high-quality, sustainable health care services.

## Figures and Tables

**Table 1:** Demographic analysis. (*n* = 650).

Variables	Number (%)
**Gender**	
Male	31 (4.8)
Female	619 (95.2)
**Age (Years)**	
20 to 30 (Generation Z)	166 (25.6)
31 to 45 (Millennials)	212 (32.6)
46 to 60 (Generation X)	272 (41.8)
**Monthly Income (Baht)**	
≤20,000	90 (13.8)
20,000 to 40,000	314 (48.3)
40,001 to 60,000	189 (29.1)
>60,000	57 (8.8)
**Monthly Income Adequacy**	
Not enough	273 (42.0)
Enough	377 (58.0)
**Years of Work Experience**	
1 to 10	346 (53.2)
11 to 20	134 (20.6)
21 to 30	104 (16.0)
31 to 40	66 (10.2)
**Working Division**	
Executives and System Administrators	32 (4.9)
Out-Patient Department	77 (11.9)
In-Patient Department	246 (37.8)
Intensive Care Unit	133 (20.5)
Special Clinic Unit	22 (3.4)
Operating Department	60 (9.2)
Anesthesia Department	42 (6.5)
Emergency Department	38 (5.8)
**Position Levels**	
Practitioner Level	396 (60.9)
Professional Level	194 (29.9)
Senior Professional Level	60 (9.2)

**Table 2:** The Minimum, Maximum, Mean ± SD, and Levels of the nurses’ UWES score. (*n* = 650).

Utrecht Work Engagement Scale	Score				Levels (*n*, %)	
	Total	Minimum	Maximum	Mean ± SD	Low	High
Vigor	42	6	42	27.90 ± 6.24	475 (73.1)	175 (26.9)
Dedication	35	5	35	24.24 ± 5.43	449 (69.1)	201 (30.9)
Absorption	42	6	42	27.29 ± 6.67	455 (70.0)	195 (30.0)

**Table 3:** Vigor-UWES levels by demographic characteristics factors. (*n* = 650).

Demographic Characteristics	Vigor-UWES Levels				*p*-Value
	Low		High		
	*n*	%	*n*	%	
**Gender**					0.493
Male	21	4.4	10	95.6	
Female	454	69.0	165	94.3	
**Age (Years)**					0.006 *
20 to 30 (Generation Z)	106	22.3	60	34.3	
31 to 45 (Millennials)	158	33.3	54	30.9	
46 to 60 (Generation X)	211	44.4	61	34.9	
**Monthly Income (Baht)**					0.033 *
≤20,000	61	12.8	29	16.6	
20,000 to 40,000	245	51.6	69	39.4	
40,001 to 60,000	133	28.0	56	32.0	
>60,000	36	7.6	21	12.0	
**Monthly Income Adequacy**					0.039 *
Not enough	211	44.4	62	35.4	
Enough	264	55.6	113	64.6	
**Years of Work Experience**					0.004 *
1 to 10	267	56.2	79	45.1	
11 to 20	99	20.8	35	20.0	
21 to 30	72	15.2	32	18.3	
31 to 40	37	7.8	29	16.6	
**Working Division**					0.067
Executives and System Administrators	18	3.8	14	8.0	
Out-Patient Department	52	10.9	25	14.3	
In-Patient Department	181	38.1	65	37.1	
Intensive Care Unit	100	21.1	33	18.9	
Special Clinic Unit	14	2.9	8	4.6	
Operating Department	42	8.8	18	10.3	
Anesthesia Department	36	7.6	6	3.4	
Emergency Department	32	6.7	6	3.4	
**Position Levels**					0.001 *
Practitioner Level	305	64.3	91	52.0	
Professional Level	137	28.8	57	32.6	
Senior Professional Level	33	6.9	27	15.4	

NOTE: * *p* ≤ 0.05.

**Table 4:** Dedication-UWES levels by demographic characteristics factors. (*n* = 650).

Demographic Characteristics	Dedication-UWES Levels				*p*-Value
	Low		High		
	*n*	%	*n*	%	
**Gender**					0.336
Male	19	4.2	12	6.0	
Female	430	95.8	189	94.0	
**Age (Years)**					<0.001 *
20 to 30 (Generation Z)	93	20.7	73	36.3	
31 to 45 (Millennials)	154	34.3	58	28.9	
46 to 60 (Generation X)	202	45.0	70	34.8	
**Monthly Income (Baht)**					<0.001 *
≤20,000	59	13.1	31	15.4	
20,000 to 40,000	240	53.5	74	36.8	
40,001 to 60,000	118	26.3	71	35.3	
>60,000	32	7.1	25	12.4	
**Monthly Income Adequacy**					0.050 *
Not enough	200	44.5	73	36.3	
Enough	249	55.5	128	63.7	
**Years of Work Experience**					<0.001 *
1 to 10	255	56.2	91	45.3	
11 to 20	100	20.8	34	16.9	
21 to 30	64	15.2	40	19.9	
31 to 40	30	7.8	36	17.9	
**Working Division**					0.108
Executives and System Administrators	15	3.3	17	8.5	
Out-Patient Department	51	11.4	26	12.9	
In-Patient Department	174	38.8	72	35.8	
Intensive Care Unit	100	22.2	33	16.4	
Special Clinic Unit	16	3.6	6	3.0	
Operating Department	41	9.1	19	9.5	
Anesthesia Department	29	6.5	13	6.5	
Emergency Department	23	5.1	15	7.5	
**Position Levels**					<0.001 *
Practitioner Level	294	65.5	102	50.8	
Professional Level	128	28.5	66	32.8	
Senior Professional Level	27	6.0	33	16.4	

NOTE: * *p* ≤ 0.05.

**Table 5:** Absorption-UWES levels by demographic characteristics factors. (*n* = 650).

Demographic Characteristics	Absorption-UWES Levels				*p*-Value
	Low		High		
	*n*	%	*n*	%	
**Gender**					0.602
Male	23	5.1	8	4.1	
Female	432	94.9	187	95.9	
**Age (Years)**					<0.001 *
20 to 30 (Generation Z)	95	20.9	71	36.4	
31 to 45 (Millennials)	151	33.2	61	31.3	
46 to 60 (Generation X)	209	45.9	63	32.3	
**Monthly Income (Baht)**					0.081
≤20,000	61	13.4	29	14.9	
20,000 to 40,000	232	51.0	82	42.1	
40,001 to 60,000	129	28.4	60	30.8	
>60,000	33	7.3	24	12.2	
**Monthly Income Adequacy**					0.231
Not enough	198	43.5	75	38.5	
Enough	257	56.5	120	61.5	
**Years of Work Experience**					<0.001 *
1 to 10	263	57.8	83	42.6	
11 to 20	92	20.2	42	21.5	
21 to 30	68	14.9	36	18.5	
31 to 40	32	7.1	34	17.4	
**Working Division**					0.063
Executives and System Administrators	16	3.5	16	8.2	
Out-Patient Department	52	11.4	25	12.8	
In-Patient Department	178	39.1	68	34.9	
Intensive Care Unit	93	20.4	40	20.5	
Special Clinic Unit	15	3.3	7	3.6	
Operating Department	37	8.1	23	11.8	
Anesthesia Department	32	7.0	10	5.1	
Emergency Department	32	7.0	6	3.1	
**Position Levels**					<0.001 *
Practitioner Level	294	64.6	102	52.3	
Professional Level	131	28.8	63	32.3	
Senior Professional Level	30	6.6	30	15.4	

NOTE: * *p* ≤ 0.05.

**Table 6:** Multivariate binary logistic analysis for Vigor-UWES levels (*n* = 650).

	Vigor-UWES Levels				
Variables	Unadjusted		Adjusted		*p*-Value
	OR	95% CI	OR	95% CI	
**Age (Years)**					
20 to 30 (Generation Z)	1		1		
31 to 45 (Millennials)	0.959	0.38–2.49	0.932	0.37–2.34	0.881
46 to 60 (Generation X)	0.717	0.29–2.36	0.780	0.28–2.20	0.639
**Monthly Income (Baht)**					
≤20,000	1		1		
20,000 to 40,000	0.488	0.28–0.85	0.478	0.28–0.83	0.009 *
40,001 to 60,000	0.390	0.19–0.83	0.414	0.20–0.87	0.019 *
>60,000	0.307	0.11–0.89	0.308	0.11–0.87	0.026 *
**Monthly Income Adequacy**					
Not enough	1		1		
Enough	1.353	0.90–2.03	1.333	0.90–1.99	0.158
**Years of Work Experience**					
1 to 10	1		1		
11 to 20	1.212	0.59–2.46	1.141	0.57–2.28	0.709
21 to 30	1.530	0.50–4.68	1.249	0.42–3.75	0.692
31 to 40	1.802	0.48–6.70	1.815	0.50–6.54	0.362
**Position Levels**					
Practitioner Level	1		1		
Professional Level	1.302	0.67–2.55	1.231	0.64–2.38	0.536
Senior Professional Level	2.620	0.89–7.64	2.106	0.77–5.79	0.149

NOTE: OR: odds ratio; CI: Confidence Interval; * *p* ≤ 0.05.

**Table 7:** Multivariate binary logistic analysis for Dedication-UWES levels (*n* = 650).

	Dedication-UWES Levels				
Variables	Unadjusted		Adjusted		*p*-Value
	OR	95% CI	OR	95% CI	
**Age (Years)**					
20 to 30 (Generation Z)	1		1		
31 to 45 (Millennials)	1.045	0.43–2.57	1.144	0.47–2.80	0.768
46 to 60 (Generation X)	0.921	0.33–2.54	0.984	0.36–2.69	0.975
**Monthly Income (Baht)**					
≤20,000	1		1		
20,000 to 40,000	0.566	0.33–0.97	0.533	0.31–0.91	0.021 *
40,001 to 60,000	0.661	0.33–1.34	0.639	0.32–1.29	0.211
>60,000	0.432	0.16–1.19	0.402	0.79–1.68	0.076
**Monthly Income Adequacy**					
Not enough	1		1		
Enough	1.171	0.79–1.72	1.149	0.79–1.68	0.475
**Years of Work Experience**					
1 to 10	1		1		
11 to 20	0.906	0.45–1.82	0.825	0.42–1.63	0.580
21 to 30	1.435	0.49–4.19	1.378	0.48–3.97	0.552
31 to 40	2.354	0.66–8.40	2.256	0.65–7.85	0.201
**Position Levels**					
Practitioner Level	1		1		
Professional Level	1.301	0.67–2.52	1.257	0.66–2.41	0.491
Senior Professional Level	2.276	0.81–6.42	2.370	0.87–6.44	0.091

NOTE: OR: odds ratio; CI: Confidence Interval; * *p* ≤ 0.05.

**Table 8:** Multivariate binary logistic analysis for Absorption-UWES levels (*n* = 650).

	Absorption-UWES Levels				
Variables	Unadjusted		Adjusted		*p*-Value
	OR	95% CI	OR	95% CI	
**Age (Years)**					
20 to 30 (Generation Z)	1		1		
31 to 45 (Millennials)	0.614	0.25–1.52	0.572	0.23–1.40	0.222
46 to 60 (Generation X)	0.4266	0.15–1.19	0.464	0.17–1.27	0.135
**Years of Work Experience**					
1 to 10	1		1		
11 to 20	1.627	0.83–3.20	1.390	0.72–2.68	0.324
21 to 30	1.630	0.54–4.89	1.072	0.37–3.08	0.898
31 to 40	2.686	0.74–9.70	1.775	0.53–5.98	0.354
**Position Levels**					
Practitioner Level	1		1		
Professional Level	0.865	0.45–1.65	0.764	0.42–1.40	0.384
Senior Professional Level	1.349	0.48–3.80	1.036	0.42–2.54	0.938

NOTE: OR: odds ratio; CI: Confidence Interval.

## Data Availability

The datasets used and/or analyzed during the current study are available from the corresponding author upon reasonable request.

## Public Involvement Statement

No public involvement in any aspect of this research.

## Guidelines and Standards Statement

No reporting guideline was used when drafting the report.
